# A Novel Multiscale Mathematical Model for Building Bone Substitute Materials for Children

**DOI:** 10.3390/ma11061045

**Published:** 2018-06-20

**Authors:** Abdennasser Chekroun, Laurent Pujo-Menjouet, Jean-Philippe Berteau

**Affiliations:** 1 Laboratoire d’Analyse Non Linéaire et Mathématiques Appliquées, University of Tlemcen, Chetouane 13000, Algeria; chekroun@math.univ-lyon1.fr; 2 Université de Lyon, Université Claude Bernard Lyon 1, CNRS UMR 5208, Institut Camille Jordan, F-69622 Villeurbanne CEDEX, France; Inria Team Dracula, Inria Grenoble Rhône-Alpes Center, 69100 Villeurbanne CEDEX, France; pujo@math.univ-lyon1.fr; 3 Department of Physical Therapy, College of Staten Island, City University of New York, New York, NY 10314, USA; 4 New York Center for Biomedical Engineering, City College of New York, City University of New York, New York, NY 10031, USA; 5 Nanoscience Initiative, Advance Science Research Center, City University of New York, New York, NY 10031, USA

**Keywords:** mathematical model, mechanical behavior, children’s bone grafts

## Abstract

Bone is an engineering marvel that achieves a unique combination of stiffness and toughness exceeding that of synthesized materials. In orthopedics, we are currently challenged for the child population that needs a less stiff but a tougher bone substitute than adults. Recent evidence suggests that the relationship between inter-molecular connections that involve the two main bone building blocks, TropoCollagen molecules (TC) and carbonated Hydroxyapatite (cAp), and bone macroscopic mechanical properties, stiffness and toughness, are key to building bone substitute materials for children. The goal of our study is to establish how inter-molecular connections that occur during bone mineralization are related to macroscopic mechanical properties in child bones. Our aim is to link the biological alterations of the TC-cAp self assembly process happening during bone mineralization to the bone macroscopic mechanical properties’ alterations during aging. To do so, we have developed a multiscale mathematical model that includes collagen cross links (TC–TC interface) from experimental studies of bone samples to forecast bone macroscopic mechanical properties. Our results support that the Young’s modulus cannot be a linear parameter if we want to solve our system. In relation to bone substitute material with innovative properties for children, our results propose values of several biological parameters, such as the number of crystals and their size, and collagen crosslink maturity for the desired bone mechanical competence. Our novel mathematical model combines mineralization and macroscopic mechanical behavior of bone and is a step forward in building mechanically customized biomimetic bone grafts that would fit children’s orthopedic needs.

## 1. Introduction

Bone is a hard–soft hybrid biomaterial that achieves an astonishing combination of stiffness, strength, and toughness that far exceeds that of currently synthesized bone substitute materials [1]. Today, most bone substitute materials are divided into non-degradable and biodegradable materials [2] and both of them present limitations. For instance, non-degradable materials need to be removed from the body, and biodegradable materials are not tailored to meet the biochemical and biomechanical requirements of the patient. While recent biodegradable materials present several combinations of polymers or ceramics with or without hydroxyapatite, none of them combine the high stiffness, high strength, and high toughness present in bone [2,3]. Furthermore, these biodegradable materials are mainly made for adult patients with orthopedic issues such as aging-related pathologies and do not provide the specific high toughness required for children bone biomechanics. Thus, reducing failure in children orthopedic issues outcomes is a great challenge in biomaterial sciences [4,5,6].

In relation to bone biomechanics, it has been well established that bone mechanical competence is a result of a tight combination of stiffness, strength, and toughness, and it comes from a highly hierarchized bone biological structure [7,8]. This arrangement consists of a hard mineral phase of carbonated Apatite (cAp) within a soft phase made of a fibrillar matrix of TropoCollagen molecule (TC) [9,10,11]. To reach this arrangement, the TC-cAp self-assembly process transforms an initial soft material made of solely collagen to a terminal hybrid hard–soft material made of approximately 60% mineral, 10–20% water, and 20–30% proteins (collagen type 1 and non-collagenous proteins (NCPs)) [12,13]. Thus, to provide new knowledge to help to synthesize bone substitute materials with mechanical properties well suited for children, it is pivotal to understand and model the link between biological components and mechanical properties and more specifically with bone toughness.

Regarding the TC-cAp self-assembly process during childhood and aging, one of the key steps is the enzymatic driven collagen assembly process where osteoblasts synthesize TC that bond two-by-two via divalent crosslinks (i.e., immature, HydroxyLysino-NorLeucine (HLNL) and DiHydroxyLysino-NorLeucine (DHLNL)). Some of the divalent crosslinks mature into trivalent CXL (i.e., mature, PYriDinoline (PYD) DeoxyPyriDinoline (DPD)) that bonds TC three by three. Then, the assembly of TC forms a collagen fibril where crosslinks provide cohesion and mechanical stability through covalent bonds [14,15,16]. In relation to the differences between adults and children regarding collagen crosslinking, child bones present a higher number of divalent crosslinks than adult bones [14,17]. Additionally, another key step of the TC-cAp self-assembly process is the mineralization process where complex chemical mechanisms enable control over crystal size, shape, orientation, phase, texture, and location. Briefly, cAp precipitates under the control of several NCPs that either nucleates or inhibits this process ( i.e., nucleators or inhibitors). Amongst all the NCPs, formation and growth of cAp are controlled by nucleators, inter alia, Bone SialoProtein (BSP) and inhibitors, inter alia, OsteoPontiN (OPN) and OsteoCalcin (OC) [18]. In relation to the differences between adults and children regarding mineralization, child bones present a higher number of small cAps than adult bones [19].

Regarding bone mechanical properties during childhood and aging, two different systems are depicted. During childhood, child bones present an increase of stiffness (increase of Young’s modulus) with a ductile behavior; during aging, adult bones present a high Young’s modulus with an increasing brittle mechanical behavior [20,21]. A long held paradigm in children bone biomechanics was to consider the relationship between bone biological components and its macroscopic mechanical properties based mainly on the link between mineral phase and macroscopic bone stiffness [22]. However, this excludes the link between soft protein phase (TC and NCP’s) and macroscopic mechanical properties. During the last decade, experimental evidence has challenged this relationship [23] and has clarified the link between the soft protein phase and bone mechanical behavior at several scales [24]. Furthermore, it has been shown that child bones present a tough mechanical behavior [17] that is correlated to the maturity of the soft collagen phase.

**Figure 1:**
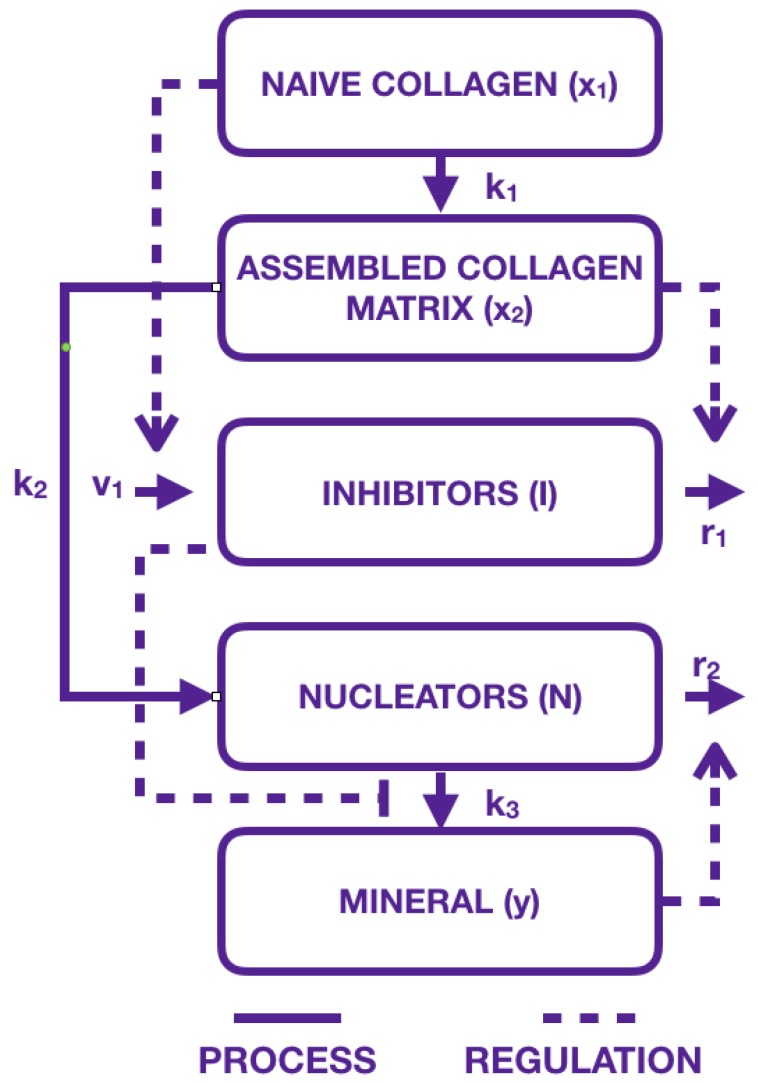
Representation of bone mineralization following Komarova et al. hypothesis in [25]. Continuous lines describe activations of the proteins involved during mineralization, while dotted lines stand for regulation.

To provide new knowledge to synthesize bone substitute materials for children, we aim to model the link between hard and soft phase, and bone mechanical properties during the TC-cAp self-assembly process. To do so, our hypothesis is to build a multiscale mathematical model that includes, on the one hand, the mechanistic understanding of the TC-cAp self assembly process (microscopic scale) and, on the other hand, the alterations of bone biomechanics during this process (macroscopic scale). In relation to the mechanistic understanding of the TC-cAp self assembly process, Komarova et al. [25] have proposed a mathematical model of mineralization that includes collagen CXL and cAp density under control of both inhibitors and nucleators. In this model, the quantity of immature CXL, $x_{1}$, independent of any outside process, linearly matures. It then reaches the $x_{2}$ population (2 first equations of system (1) corresponding to the 2 first steps of (Figure 1)).

(1) $$\left\{\begin{array}{cccc} x_{1}^{\prime } & = & - & k_{1}x_{1},\\ x_{2}^{\prime } & = & & k_{1}x_{1},\\ I^{\prime } & = & & v_{1}x_{1}-r_{1}x_{2}I,\\ N^{\prime } & = & & k_{2}x_{2}^{\prime }-r_{2}y^{\prime }N,\\ y^{\prime } & = & & k_{3}bN\setminus \left(b+I^{a}\right). \end{array}\right.$$

The variations of both of these CXLs imply changes in the variation of the inhibitor population (*I* in the third equation of the system (1) that tends to decrease when immature CXL increases. Nucleator (*N*) production then depends on the variation of mature CXL (and indirectly on immature through the second equation, and inhibitor through the fifth equation in the system (1)). However, their model did not include any relationship with mechanical properties of the bone, mineral size, or the OC–OPN complex. In relation to the alterations of bone biomechanics during this process, Ott et al. [26] have proposed the use of the Ramberg–Osgood power law to approximate mechanical data of stiff and tough bone behavior. They have optimized their experimental data to obtain the two material parameters *n* and *k* in the Ramberg–Osgood power law (2) for each measured stress–strain (i.e., ε,σ) curve obtained from experimental tensile tests of bone samples.

(2) $$\varepsilon =\frac{\sigma }{E}+k.{\left(\frac{\sigma }{E}\right)}^{n}.$$

The goal of our study is to establish how intermolecular connections affect macroscopic mechanical properties in bones by proposing a mathematical model that links the TC-cAp self-assembly process and mechanical properties using experimental data that we previously published [17]. We propose a mathematical model describing the biological interaction of TC-cAp, where we include collagen cross links (TC–TC interface) and cAp density from experimental studies on children and adults bone (1). We also take into account the correlative phenomenon with mechanical properties integrated in a Ramberg–Osgood power law (2). Our model is to be considered as a first step to forecast mechanical properties for bone substitute materials for the child population.

## 2. Model and Results

### 2.1. Modeling the Biological Components: An Inspirational Model

Our model is inspired by Komarova et al. [25]. They proposed a model that evolves over time where collagen maturation is described by immature collagen matrix ($x_{1}$) maturing to collagen matrix ($x_{2}$). Mineral is described by Hydroxyapatite (*y*), the main mineral in bone. Interaction between mineral and collagen maturation is modeled, on the one hand, by Inhibitors (*I*), which are molecules inhibiting mineralization, and, on the other hand, by Nucleators (*N*), initiating the mineral precipitation. Their general model given in System (1) and represented in Figure 1 is given with specific values of parameters to fit data (see [25]) by

(3) $$\left\{\begin{array}{cccc} x_{1}^{\prime }\left(t\right) & = & - & 0.1x_{1}\left(t\right),\;\\ x_{2}^{\prime }\left(t\right) & = & & 0.1x_{1}\left(t\right),\;\\ I^{\prime }\left(t\right) & = & & 0.1x_{1}\left(t\right)-0.2x_{2}\left(t\right)I\left(t\right),\;\\ N^{\prime }\left(t\right) & = & & x_{2}^{\prime }\left(t\right)-12y^{\prime }\left(t\right)N\left(t\right),\;\\ y^{\prime }\left(t\right) & = & & 0.001N\left(t\right)\setminus (0.001+I^{10}\left(t\right))\; \end{array}\right.$$

at any time t≥0. In the following, we keep the parameters values estimated by Komarova et al. to allow comparisons with their results.

### 2.2. Our Mathematical Model

To link biology with mechanics, our aim is to use experimental data that correlate biological parameters to mechanical parameters. Thus, in the Komarova et al. model that describes the interactions between mineral, inhibitors, nucleators, and collagen maturity, we have added a relationship between collagen maturity and mechanical behavior that was depicted by Berteau et al. [17]. They found a significant linear relationship between the plastic energy dissipated before rupture (i.e., $\omega_{p}$) and the ratio of collagen maturity (i.e., $x_{1}\left(t\right)/x_{2}\left(t\right)))$ that is depicted by the following equation:

$$\frac{x_{1}\left(t\right)}{x_{2}\left(t\right)}=0.71\omega_{p}\left(t\right)+1.$$

This linear relationship shows that collagen with more immature cross-links is more likely to plastically deform before fracture. Since broken inter-fibrillar cross-links help dissipate energy, it could be tempting to speculate that the immature cross-links broke later than the mature crosslinks, as has recently been suggested by numerical simulations [27]. However, this relationship does not mean causality between the two parameters, thus it has to be used as a link between these two parameters as a function of the age of the tissue (i.e., time (t)). Thus, the major contribution consists in the values being separated into two groups: the children, with $\omega_{p}$ above 2 MPa and with a ratio below 2, and the adults, with $\omega_{p}$ below 2 MPa and with a ratio above 2 [17]. It suggests that cortical bone samples with a ratio above 2 have a greater capacity for plastic deformation (higher toughness) and that conversely, cortical bone samples with a ratio lower than 2 are unable to plastically deform (low toughness).

In relation to this ratio of collagen maturity, it has been written in the Komarova system as a transfer of collagen from a compartment $x_{1}$ to a compartment $x_{2}$. While this is quite efficient for a simple model, we believe that this could be more precisely described as an effective evolution in maturity with an age structured partial differential equation. To do so, let us consider a population of collagen c(t,a) at a given time t≥0 with age *a*, where age is considered here as the crosslink interaction with collagen. In other words, c(t,a) would correspond to all collagens with the same level of maturity, which can range from no crosslinks at all (a=0) to all collagen proteins connected by crosslinks (a very large value of *a*). For convenience (technical reasons), we set *a* in the interval [0,+∞). We consider that immature collagen crosslinks (also called naive collagens by Komarova et al. in [25]) are the ones where “age” is less than equal to a certain positive value, α>0. That is c(t,a) for a<α stands for the naive collagen, while c(t,a) for a>α describes the mature collagen or assembled collagen matrix as mentioned by Komarova et al. Therefore, integrating from 0 to α gives the total population of immature collagen crosslinks and is denoted by $C_{1}\left(t\right)$, with

$$C_{1}\left(t\right)=\int_{0}^{\alpha }c\left(t,a\right)da,$$

and corresponds to the $x_{1}\left(t\right)$ in [25]. On the other hand, integrating from α to *∞* gives the total population of mature collagen crosslinks. It is denoted by $C_{2}\left(t\right)$, with

$$C_{2}\left(t\right)=\int_{\alpha }^{+\infty }c\left(t,a\right)da$$

corresponding to $x_{2}\left(t\right)$ in [25].

The total population of all collagen crosslinks is then given by

$$C\left(t\right)=\int_{0}^{+\infty }c\left(t,a\right)da=C_{1}\left(t\right)+C_{2}\left(t\right)$$

and would correspond to $x_{1}\left(t\right)+x_{2}\left(t\right)$. Evolution of the maturity of the whole collagen crosslink population, denoted by F(t), is then given by the ratio of mature collagen crosslinks $C_{2}\left(t\right)$ over the total quantity of collagen crosslinks C(t) at time *t*. It is given by

$$F\left(t\right)=\frac{C_{2}\left(t\right)}{C(t)}.$$

Note that F(t) is an increasing function from 0 to 1. Since no collagen crosslinks are created or degraded during our experiments, we consider the total population constant in time, that is C(t)=K, and the evolution of population c(t,a) is given by the simple partial differential equation, for t>0 and a∈(0,+∞)

(4) $$c_{t}\left(t,a\right)+c_{a}\left(t,a\right)=0.$$

This equation is completed by the following boundary condition, for t>0,

(5) c(t,0)=0

since no new collagen crosslinks is formed.

Initial conditions, for a∈(0,+∞), are

(6) $$c\left(0,a\right)=c_{0}\left(a\right),$$

where $c_{0}\left(a\right)$ is the initial distribution of collagen at time t=0. This function is given. It should be a continuous function that has different shapes depending on the experiments. That is, all collagen crosslinks are immature at the beginning or can be equally distributed in age. This part entirely depends on the experimental hypothesis at time t=0.

On the other hand, we believe that mineralization considered in [25] needs a more precise treatment than just an evolution of minerals *y* (see System (1)). From what has been mentioned in the introduction, mineralization consists of complex mechanisms leading to both precipitate cAp and control crystal size under the control of inhibitors and nucleators. It seems then quite natural to describe evolution of minerals as an aggregation model. Minerals are then denoted by m(t,x) at time t>0 with size *x*. For technical reasons, we assume s∈[0,+∞). Minerals can grow in size, with their growth velocity controlled by collagen population maturity F(t). Furthermore, their aggregation rate is assumed to be controlled both by inhibitors I(t) and nucleators N(t). The way they are controlled is depicted in terms such as growth velocity and aggregation terms.

Regarding growth velocity, we assume that, when the collagen crosslink population is very immature, that is F(t) is close to 0, velocity V(F(t)) is very small, increases to a maximum value, and thendecreases again when the collagen population is mostly mature to become 0 when F(t)=1 (the whole collagen crosslink population is mature), that is V(1)=0. The growth process then stops naturally.

Regarding aggregation terms, we decided to follow the rule given by Komarova et al., which is increasing with increased nucleators population and decreasing with increased inhibitors population.

Thus, the growth and aggregation mineral model is given by the following size structured partial differential equation

(7) $$\begin{array}{c} m_{t}\left(t,x\right)+V\left(F\left(t\right)\right)m_{x}\left(t,x\right)=\;\\ \;k\left(F\left(t\right)\right)\int_{0}^{x}m\left(t,x-y\right)m\left(t,y\right)dy-2k\left(F\left(t\right)\right)m\left(t,x\right)\int_{0}^{+\infty }m\left(t,y\right)dy. \end{array}$$

The left hand side of Equation (7) describes the evolution in time and size of the crystal population. The first term on the right hand side of Equation (7) indicates that a new mineral of size *x* results from the joining of two smaller minerals of lengths x-y and *y*. The second term on the right hand side of Equation (7) describes the loss of a mineral of length *x* when it joins with another mineral of any length to create a larger mineral. Furthermore, symmetry requires the factor 2 because we hypothesize a one-dimensional growth. Additionally, this equation needs an initial condition given for x∈(0,+∞) by

(8) $$m\left(0,x\right)=m_{0}\left(x\right)$$

where $m_{0}\left(x\right)$ is the initial distribution of minerals at time t=0. Similar to the collagen crosslinks equation, this function is a continuous function that has different shapes depending on the experiments.

Regarding the precipitate formation of cAP, we consider that this event is regulated both by nucleators and inhibitors with the following boundary condition for the minerals, for t>0:

(9) $$m\left(t,0\right)=\frac{0.001N(t)}{\left(0.001+I^{10}\left(t\right)\right)}\exp\left(-\eta M\left(t\right)\right).$$

**Figure 2:**
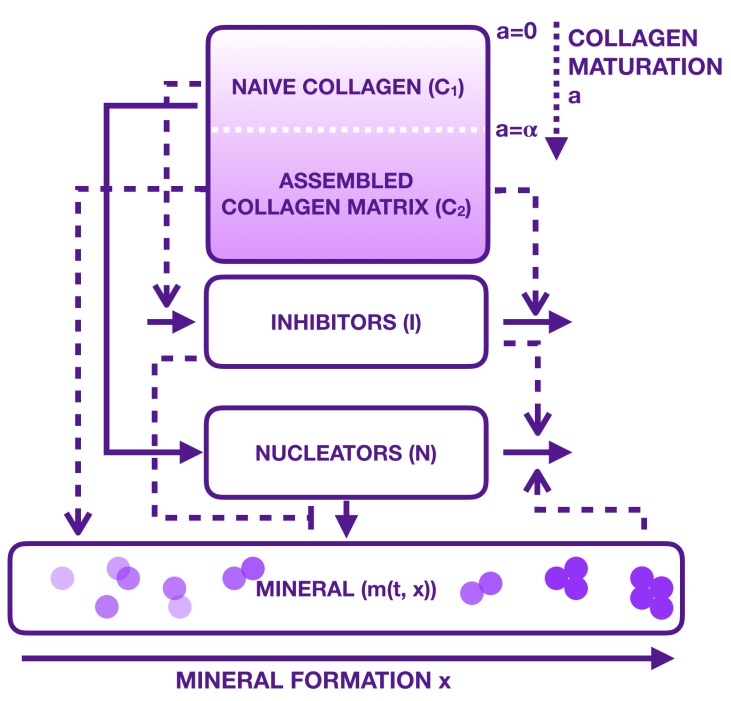
Representation of bone mineralization following the Komarova et al. hypothesis in [25] with modifications: the collagen is not considered to be in two compartments but in a continuous maturity evolution (immature a∈[0,α), to mature (α,+∞)). Mineralization is supposed to precipitate (size 0) to crystals (size x>0). Aggregation is represented by the purple “balls” as a very schematic representation of crystal forming. Continuous lines describe activations of the involved during mineralization, while dotted lines stand for the regulation.

Thus, our new model consisting of Equations (4)–(9) is complete. This new model can be illustrated by a modified version of the scheme (Figure 1) by Figure 2. In this figure, collagen and mineral compartments are modified, whereas the influence of inhibitors and nucleators remain the same:

- collagen compartments $x_{1}$ and $x_{2}$ have been replaced by a single compartment where immature collagen crosslinks progressively moves toward the assembled collagen matrix;
- mineral formation is created from precipitated crystal growing in function of collagen and aggregating in function of inhibitors and nucleators.

This is also given by the following system adapted from System (3) with our new model as described in the introduction and the experimental relationship [17], for t>0:

(10) $$\left\{\begin{array}{c} I^{\prime }\left(t\right)=0.1C_{1}\left(t\right)-0.2C_{2}\left(t\right)I\left(t\right),\;\\ N^{\prime }\left(t\right)=0.1C_{1}\left(t\right)-\frac{0.012N^{2}\left(t\right)}{\left(0.001+I^{10}\left(t\right)\right)},\;\\ \omega_{p}^{\prime }\left(t\right)=\frac{-0.1C_{1}\left(t\right)C_{2}\left(t\right)-0.1C_{1}^{2}\left(t\right)}{0.71C_{2}^{2}\left(t\right)}. \end{array}\right.$$

We propose our new model as an improvement of the one introduced by Komarova et al. However, ours is more complex, so we decided not to analyze the system of partial differential equations or the asymptotic behavior of the distributions c(t,a) and m(t,x) when *t* goes to infinity. Our objective here was to compare our model to the one proposed by Komarova et al. and to extend it to bone macroscopic mechanical properties (multiscale modeling). For this purpose, we have integrated the system with respect to the structure variables *a* and *x* and analyzed the coupled system of non-linear differential equations. This is developed in Section 3.1.

### 2.3. Results

**Figure 3:**
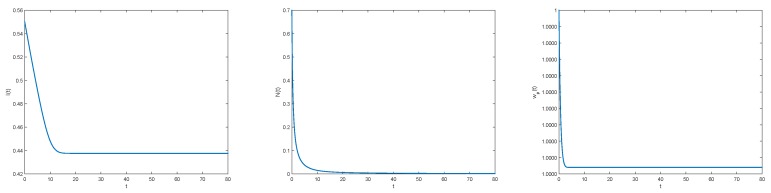
Evolution of I(t) (**Left**), N(t) (**Middle**), and $w_{p}\left(t\right)$ (**Right**) with I(0)=0.55, N(0)=0.7, and $w_{p}\left(0\right)=1$.

Using functions *V* et *k* given in Section 3.2, straightforward numerical simulations give qualitative behavior of inhibitors I(t), nucleators N(t), and function $\omega_{p}$ in Figure 3.

**Figure 4:**
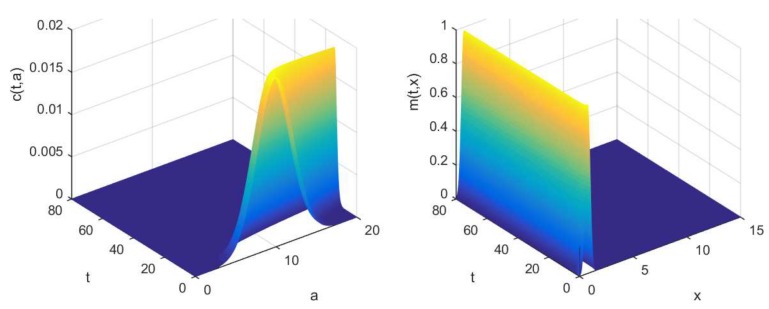
(**Left**) Population of minerals m(t,x). (**Right**) Evolution of collagen c(t,a). Parameters are α=8, η=0.2.

**Figure 5:**
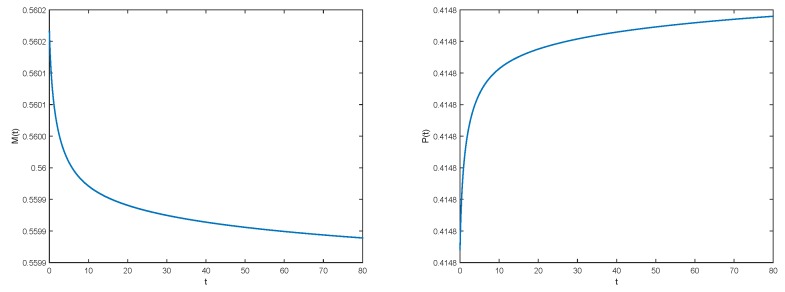
Evolution of minerals of all sizes $M\left(t\right)=\int_{0}^{+\infty }m\left(t,x\right)dx$ (**Left**) and the total mineral mass $P\left(t\right)=\int_{0}^{+\infty }xm\left(t,x\right)dx$ (**Right**).

Evolutions of collagens c(t,a) and m(t,x) are represented in Figure 4, while evolutions of total population M(t) and P(t) are shown in Figure 5.

**Figure 6:**
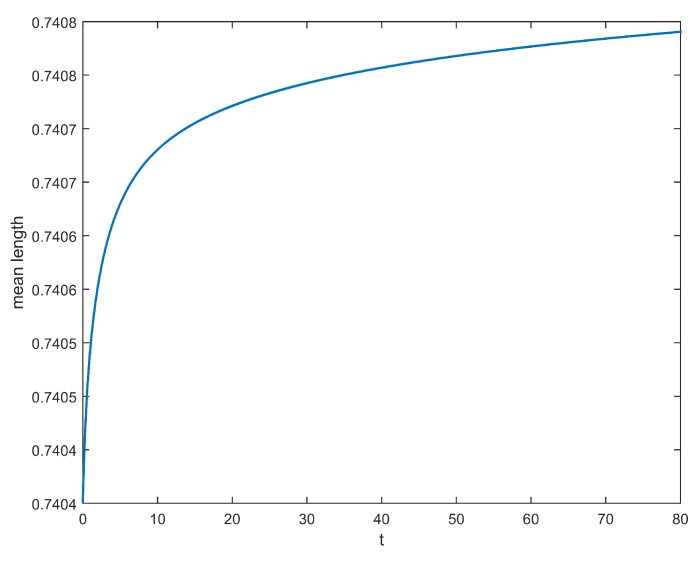
Evolution of the crystal mean size P(t)/M(t) .

It is quite straightforward to see the evolution of crystal mean size in time (see Figure 6). It is clear to see that this mean size reaches a threshold at equilibrium.

### 2.4. Multiscale Modelling

For the numerical illustration of the qualitative behavior of macroscopic mechanistic properties of the bone depending on the molecular dynamics of its structure, we have to set up a relationship between the two scales. In other words, the parameters *n* and β of Equation (2) of model from Ott et al. need to be linked as follows:

$$\epsilon =\frac{\sigma }{E}+\beta {\left(\frac{\sigma }{E}\right)}^{n},$$

with n=n(F(t),M(t)) non-decreasing with respect to *F* and non-increasing in function of *M*. The term β=β(F(t),M(t)) on the other hand is a non-increasing function of *F* and non-decreasing with respect to *M*. For instance, *n* and β are suggested here to be given by

$$n\left(F\left(t\right),M\left(t\right)\right)=30{\left(F\left(t\right)\right)}^{10}-0.1\left(M\left(t\right)\right)$$

and

$$\beta \left(F\left(t\right),M\left(t\right)\right)=-0.5{\left(F\left(t\right)\right)}^{5}+0.85M\left(t\right).$$

**Figure 7:**
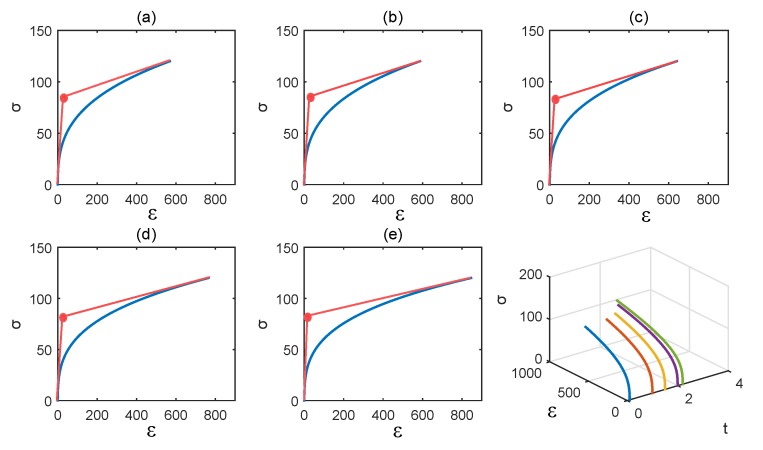
Parameters are E=10, α=8, and η=0.2. The values of the parameter *t* are as follows: (**a**) t=0.1; (**b**) t=1; (**c**) t=1.5; (**d**) t=2; (**e**) t=2.2.

A table that contains the different values of *F* and *M* by varying *t* is given in Table 1 below. These values have been used in each graph (from (a) to (e)) of Figure 7 which corresponds to the different time points (from 0.1 to 2.2). The last graph (bottom right) of Figure 7 summarizes all these representations in one.

Finally, the term E=E(F(t),M(t)) is described as a non-increasing function of *F* and the non-decreasing function of *M*. For instance, *E* can be taken as

$$E\left(F\left(t\right),M\left(t\right)\right)=-5{\left(F\left(t\right)\right)}^{2}+10M\left(t\right).$$

## 3. Materials and Methods

### 3.1. An Associated System of Ordinary Differential Equation

By using the characteristics method for (4), we obtain the following expression of *c*

(11) $$c\left(t,a\right)=\left\{\begin{array}{cc} c(t-a,0)=0, & \mathrm{for}\;t>a,\;\\ c\left(0,a-t\right)=c_{0}\left(a-t\right),\; & \mathrm{for}\;t\leq a. \end{array}\right.$$

Let us denote, for t>0,

$$C\left(t\right):=\int_{0}^{+\infty }c\left(t,a\right)da,\;M\left(t\right):=\int_{0}^{+\infty }m\left(t,x\right)dx\;\mathrm{and}\;P\left(t\right):=\int_{0}^{+\infty }xm\left(t,x\right)dx.$$

Both C(t) and M(t) are the total populations of collagen and mineral, respectively, and P(t) corresponds to the mass of mineral in the bone we study. The expression (11) leads, for t>0, to

$$C\left(t\right)=\int_{0}^{t}c\left(t-a,0\right)da+\int_{t}^{+\infty }c_{0}\left(a-t\right)da=\int_{t}^{+\infty }c_{0}\left(a-t\right)da.$$

We integrate Equation (7) over the size variable *x*. Moreover, we take into account that $\lim_{x\mapsto +\infty }m\left(t,x\right)=0$, which means that there does not exist any minerals of infinite size, and seems biologically reasonable. We obtain, for t>0,

$$\begin{array}{c} M^{\prime }\left(t\right)=k\left(N\left(t\right),I\left(t\right),F\left(t\right)\right)\int_{0}^{+\infty }\int_{0}^{x}m\left(t,x-y\right)m\left(t,y\right)dydx+V\left(F\left(t\right)\right)m\left(t,0\right)\;\\ \;-2k\left(N\left(t\right),I\left(t\right),F\left(t\right)\right)\int_{0}^{+\infty }m\left(t,x\right)\int_{0}^{+\infty }m\left(t,y\right)dydx. \end{array}$$

Thanks to the Fubini’s theorem, we obtain, for t>0,

$$\int_{0}^{+\infty }\int_{0}^{x}m\left(t,x-y\right)m\left(t,y\right)dydx=\int_{0}^{+\infty }m\left(t,y\right)\int_{y}^{+\infty }m\left(t,x-y\right)dxdy=M^{2}\left(t\right).$$

Then,

$$M^{\prime }\left(t\right)=-k\left(N\left(t\right),I\left(t\right),F\left(t\right)\right)M^{2}\left(t\right)+V\left(F\left(t\right)\right)\frac{0.001N(t)}{\left(0.001+I^{10}\left(t\right)\right)}e^{-\eta M(t)},\;t>0.$$

The equation of P(t) is obtained as above by multiplying Equation (7) by the size variable *x* and then integrate it over *x*. We obtain, for t>0,

$$\begin{array}{c} P^{\prime }\left(t\right)=V\left(F\left(t\right)\right)M\left(t\right)-2k\left(N\left(t\right),I\left(t\right),F\left(t\right)\right)P\left(t\right)M\left(t\right)\;\\ \;+k\left(N\left(t\right),I\left(t\right),F\left(t\right)\right)\int_{0}^{+\infty }x\int_{0}^{x}m\left(t,x-y\right)m\left(t,y\right)dydx. \end{array}$$

We can compute the nonlocal term and we obtain, for t>0,

$$\begin{array}{ccc} \int_{0}^{+\infty }x\int_{0}^{x}m\left(t,x-y\right)m\left(t,y\right)dydx & = & \int_{0}^{+\infty }\int_{y}^{+\infty }xm\left(t,x-y\right)m\left(t,y\right)dxdy,\;\\ & = & \int_{0}^{+\infty }\int_{0}^{+\infty }\left(s+y\right)m\left(t,s\right)m\left(t,y\right)dsdy,\;\\ & = & 2M(t)P(t). \end{array}$$

Therefore, the equation of P(t) becomes

$$P^{\prime }\left(t\right)=V\left(F\left(t\right)\right)M\left(t\right),\;t>0.$$

### 3.2. Numerical Illustrations

Our aim is only to study our model in a qualitative way and compare it with the model given in [25]. This is why we keep parameters as close as possible to the ones used by those authors. However, we had to choose growth velocity function *V* to have the properties given in the previous section, that is equal to 0 when s=0, then increasing to a certain threshold, then decreasing when *s* is equal to 1. We chose the following function:

$$V\left(s\right):=\frac{s}{50}e^{-0.5{\left(s+13\right)}^{2}},\;s\geq 0.$$

For function *k*, influence from inhibitors and nucleators are as follows: inhibitors are supposed to decrease aggregation, while nucleators increase them. We describe it with the following formulation:

$$k\left(N,I,s\right):=\frac{10N}{1+I^{2}}se^{-10s^{2}+s},\;s\geq 0.$$

**Figure 8:**
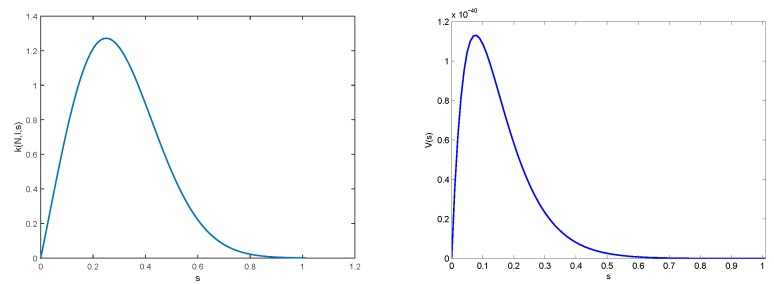
The graph of functions s↦k(N,I,s) (**Left**) and s↦V(s) (**Right**). The graph of function s↦k(s,N(1),I(1)) was plotted at time t=1, that is N(1)=0.2010 and I(1)=0.5426.

Finally, values for maturation threshold is chosen to be α=3 and the value of parameter η=0.2. Representations of function *V* and *k* are given in Figure 8.

## 4. Discussion

In this study, we have developed a new model of bone biomechanics for bone substitute materials based on (i) a mechanistic model of bone mineralization and on (ii) a mechanical law of elasto-plastic material. We have tested the ability to combine (i) a mathematical model of bone mechanics with (ii) a model of bone biology by using an experimental relationship from our previous study [17]. We combined (i) a model of matrix biomineralization based on a mechanistic understanding of the interactions between the maturity of the collagen matrix and nucleators/inhibitors of mineralization with (ii) a model of bone mechanics based on a law for elasto-plastic material by using an experimental law between plastic energy and collagen maturity. Here, we present a first qualitative approach to determine the key factors of mineralization of the bone matrix for building bone substitute with customized mechanical properties for the child population. Our results show that we have been able to combine two different models to propose a mathematical model of bone biomechanics. Furthermore, we have been able to describe the mechanical behavior of bone samples at several steps of bone matrix maturation with only a few parameters. Indeed, our results present differences in strain/stress curves based on the biomineralization of the matrix at the molecular scale and in qualitative indications for the values of the collagen, the mineral, and the inhibitors and nucleators of mineralization in each case.

**Figure 9:**
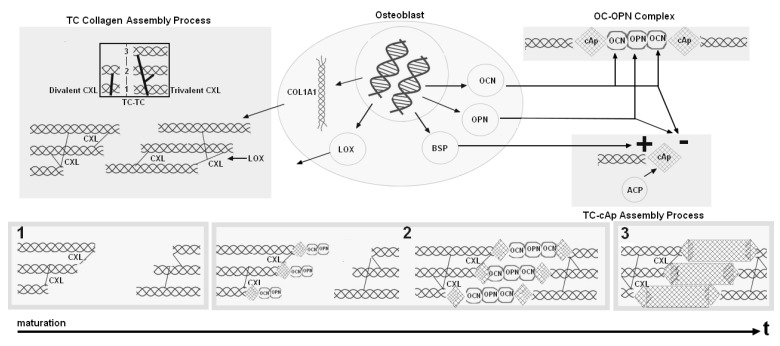
Three states of mineralization of the collagen fiber that we modeled using the interactions between collagen crosslink maturity, inhibitors, nucleators and cAp precipitation. Step 1 corresponds to non-mineralization where we found only divalent CXL in the collagen fibril, no cAp minerals, and no OPN–OC complex. Step 2 is the intermediate mineralization where we found mainly divalent CXL in the collagen fibril, cAp minerals, and an OPN–OC complex between the minerals. Step 3 is the late stage of mineralization where we found large aggregated crystals and no OPN–OC complex.

Regarding the interfacial interactions of TC-cAp that we modeled, they can be depicted in three different steps of mineralization of the collagen fibril and are presented on Figure 9.

For decades, hydroxyapatite has been the most common material for building bone substitute. Furthermore, mineral density has been the gold standard for evaluating bone strength in clinical practice. In our study, our results show that the evolution of both mineral size and bone mass presents the clearest alterations over time (Figure 6), especially from the immature stage to the mature stage. Thus, in the goal of building bone substitutes for children, our results support that the mineral phase remains one of the main parameters for mimicking bone strength. Regarding the inhibitors of mineralization such as OC and OPN, their level is very high at the start of maturation, but their concentration decreases when minerals reach maturity. Furthermore, our model shows that inhibitors present a baseline in the model, suggesting that during mineralization there is always a secretion of inhibitors. Regarding the nucleators, our results show an extremely fast drop compared to inhibitors. Taken together, this suggests that mineral properties are more related to inhibitor alterations than to nucleator alterations. However, it is difficult to compare the alterations of our values to experimental values because most of the models used in experimental studies come from knock-out animals where either nucleators or inhibitors are completely shut down. Our results are the first to present in silico data of the alterations of nucleators, inhibitors, and mineral properties during bone maturation. Furthermore, our results suggest a key role of inhibitor alterations during bone matrix maturation.

**Figure 10:**
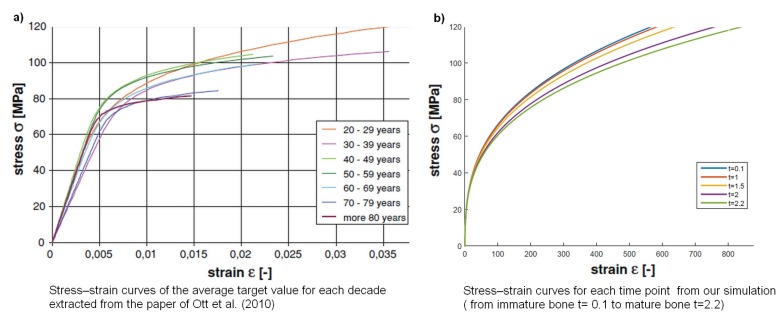
Stress–strain curves from (**a**) Ott et al. [26] and (**b**) our curves.

Regarding the mechanical properties, our model shows differences in bone mechanics based on the maturity (age) of the matrix that are similar to Ott et al. [26]. For instance, our results show that, in the case of an immature matrix and for the same modulus of elasticity (fixed here at 10 GPa), the ultimate strain (fixed at 120 MPa) is reached earlier in an immature case that in a mature case. This supports the data of Ott et al. [26], where the curves for the young decades reached a higher ultimate strain faster than the ones for the older decades (Figure 10). However, we did not include a criterion of rupture making it difficult to describe the mechanical behavior after this ultimate strain. Regarding the yield point (which describes the limits between elasticity and plasticity), we decided to use the same method as Ott et al. [26]. However, we have not been able to distinguish clear differences for the yield point between the conditions tested (Figure 7). This method is different from the one commonly used in experimental mechanics (offset method at 0.2%) making it difficult to clearly find the limit between elastic and plastic behavior in our data. Thus, further simulations are needed to be able to compare the two methods of calculation of the yield point, and to compare our results against our experimental data. Regarding the toughness, we consider toughness as the sum of elastic energy and plastic energy (i.e., $w_{e}+w_{p}$). Our results show an increase of toughness with age that is not supported by experimental data. This may come from the lack of criterion of rupture and our method of yield point assessment. Thus, we cannot draw conclusions on the toughness data obtained here.

Regarding elasticity, and more specifically Young’s modulus (*E*) obtained here, a constant *E* value for the elastic part shows our inability to solve the system in a realistic way. Most of the time, it is believed that *E* is constant and related to the mineral density of the bone. Our results suggest that, if we want to determine a yield point in our curve, we have to use a non-linear E. Thus, we used a non-linear *E*, and we obtained more realistic curves with a more defined yield point for each of them. One of our main issues though, which is the topic of our future work, is determining the most appropriate non-linear function with adjusted parameters based on experimental data and mathematical methods to estimate them (such as optimization (least square or gradient descent) and kriging methods). These remarks also apply to non-linear functions *k* and *V*, elaborated from a mechanical hypothesis with the least phenomenological assumptions possible. Our results reject the common idea of a bone that is linear-elastic. Consequently, this behooves us to improve the Ramberg–Osgood power law used here by including a Kelvin–Voigt model. This consists of a linear elastic spring and a linear viscous dash-pot connected in parallel and could be related to the OC–OPN complex. The spring model with the instantaneous bond deformation of the material, and its magnitude, is related to the fraction of mechanical energy stored reversibly as strain energy commonly used to model the creep behavior of polymers with the following equation:

(12) $$\sigma =E\varepsilon +\eta \dot{\varepsilon },$$

where σ and ε are analogous to the spring force and displacement, σ is the stress applied, *E* is the stiffness of the spring element therefore has units of $N/m^{2}$, ε is the strain of the spring element, η is the viscosity of the material, and $\dot{\varepsilon }$ is the strain of the dash-pot element.

Furthermore, it seems pivotal to consider simple elements for modeling the connections between buildings blocks at the Interfibrillar interfaces. For instance, it is generally believed that CXL transfers force from one molecule to another. Here, we did not take into account the sliding between the TC molecules. One of the most commonly rheological models used for CXL has been proposed by Uzel et al. [28]. From in silico experimentation, the authors proposed that the contribution of CXL can be modeled through an element called “delay.” They combined this element to two others: an elastic element for the tensile stiffnesses and a frictional one for the intermolecular slippage.

The biomechanics of bone substitute material is more complex than the Ramberg–Osgood power law used here. While this law has been used as a base to determine the individual fracture risk for patients without direct mechanical measurement of their bone [26], the authors have integrated the mechanics of lower scales. Indeed, by taking into account age and gender, microstructure and geometry of the bone they can calculate the tolerable loads and compare them with the age-dependent averages, offering a new chance to detect osteoporosis. When dealing with child population, the issue is different and relatively new in the field of biomaterials. Indeed, we have to take into account the maturity of all the parameters before bone reaches its full maturity. Furthermore, each maturity step impacts bone mechanics. For instance, during the TC-cAp self-assembly process, trivalent CXLs are considered stiffer than divalent CXLs, but both provide cohesion and mechanical stability to the fibrils. Furthermore, the presence of cAp significantly enhances elasticity under the dependence on their mineral crystal thickness. Regarding NCPs, it has been recently suggested that an OPN–OC complex is created between two cAps and acts as dilatation bond that might be linked to the bone visco-elasticity. All of these elements vary with age and are challenging to model during human growth.

Our ultimate goal is to develop a biomechanics law to predict bone substitute mechanical properties based on experimental data. Applying the use of this model to daily medical/scientific life, our model aims to provide the specific toughness required for building a customized bone substitute for a child. Indeed, by knowing the degree of collagen crosslink maturity that can be obtained by urine analysis in children, it might be possible to predict the overall macroscopic behavior of their bones. This could then indicate the quantity of bone toughness that children need for their bone substitutes. This could also be achieved by knowing the crystal size of the bone that the medical team aim to replace. To do so, in vivo Raman spectroscopy for bone assessment is under development to provide bone crystal size [29], and our model could predict the mechanical properties needed for the bone substitute. The work presented here is a milestone on the road to our ultimate goal, and it lays the groundwork to predict bone mechanical behavior from only one of the parameters we entered in the model.

## 5. Conclusions

Our new model links bone matrix mineralization and bone mechanical properties at the macroscopic level. Our results show the feasibility of linking these two systems with a multiscale approach and by taking into account a simple modeling hypothesis. Furthermore, our results support that the Young’s modulus cannot be a linear parameter if we want to solve our system. This finding supports that bone elasticity is not a linear phenomenon. In relation to bone substitute material with innovative properties for children, our results propose values of several biological parameters, such as the number of crystals and their size and the collagen crosslink maturity from the desired bone mechanical competence. Thus, our model lays the groundwork to provide new knowledge for building bone substitute material for the child population.

References

## Figures and Tables

**Table 1:** Table of parameters used for our numerical simulations.

*t*		F(t)		M(t)	Elastic Energy + Plastic Energy
0.1		0.7960		0.5598	5.0901
1		0.7964		0.5576	5.2620
1.5		0.7974		0.5569	5.7648
2		0.7993		0.5565	6.9175
2.2		0.8004		0.5563	7.6916
